# The Safety and Efficacy of Camrelizumab and Its Combination With Apatinib in Various Solid Cancers

**DOI:** 10.3389/fphar.2020.568477

**Published:** 2020-10-21

**Authors:** Kunlun Wang, Bingxu Li, Mengxi Li, Shenglei Li, Hui Yang, Ling Yuan

**Affiliations:** Department of Radiation Oncology, The Affiliated Cancer Hospital of Zhengzhou University, Zhengzhou, China

**Keywords:** camrelizumab, apatinib, safety, efficacy, solid cancers, meta-analysis

## Abstract

**Background**: Camrelizumab (SHR1210) is a high-affinity, humanized immunoglobulin programmed cell death 1 (PD-1) monoclonal antibody. It was developed by Jiangsu Hengrui Medicine Co. Ltd. and has been approved for relapsed or refractory classical Hodgkin lymphoma patients and hepatocellular carcinoma patients in China. Apatinib is an orally administered vascular endothelial growth factor receptor-2 (VEGFR-2) tyrosine kinase inhibitor and has been approved for advanced gastric adenocarcinoma or gastroesophageal junction adenocarcinoma in China. Camrelizumab alone and its combination with apatinib have been used in the treatment of various solid cancers.

**Methods**: We searched Embase, PubMed, and other databases with the keyword “camrelizumab” or “SHR1210,” and evaluated the safety and efficacy data of the involved studies. Adverse events (AEs) mentioned in at least two studies were summarized, including any grade and grade ≥3 treatment-related AEs. Meanwhile, efficacy data were collected, such as overall response rate (ORR), disease control rate (DCR), duration of response, 6-month progression-free survival (PFS) rate, median PFS time, 12-month overall survival rate, and median overall survival time.

**Results**: The major AEs of camrelizumab alone were reactive cutaneous capillary endothelial proliferation, fatigue, aspartate aminotransferase increase, proteinuria, pruritus, and alanine transaminase increase. The ORR and DCR were 20.2% (95% CI: 15.1–26.6%, p = 0.000, I^2^ = 70.360) and 45.8% (95% CI: 39.0–52.7%, p = 0.256, I^2^ = 58.661), respectively. In the three studies of combination therapy, two studies were combined with apatinib and one combined with chemotherapy. For these studies, common AEs were hypertension, platelet count decrease, nausea, proteinuria, aspartate aminotransferase increase, and white blood cell count decrease. The pooled ORR, DCR, and 6-month PFS rate were 41.8% (95% CI: 29.7–54.9%, p = 0.220, I^2^ = 86.265), 82.4% (95% CI: 75.9–87.4%, p = 0.000, I^2^ = 55.207), and 56.2% (95% CI: 35.8–74.6%, p = 0.559, I^2^ = 79.739), respectively.

**Conclusion**: Camrelizumab and its combination are tolerable and appear to be efficient in treating numerous solid cancers. The combination therapy appears to have better efficacy with durable toxicity. However, these remain to be shown in future studies. Besides, baseline lactate dehydrogenase, programmed cell death ligand 1 (PD-L1) expression, tumor mutation burden, and the incidence of reactive cutaneous capillary endothelial proliferation may be efficacy predictors and need to be clarified in further studies.

## Introduction

Immune checkpoint inhibitors (ICIs) targeting the programmed death 1/programmed death ligand 1 (PD-1/PD-L1) pathway have become the hottest therapeutics for various tumors. Tumor cells are able to escape immune surveillance through the interaction between immune checkpoints and their ligands (Pardoll 2012). Inhibitors targeting these pathways could break immunity evasion, enhance antitumor immunity, and produce durable clinical responses (Sharma and Allison, 2015). A lot of PD-1 or PD-L1 inhibitors have been developed and showed promising efficacy results.

Camrelizumab (SHR1210, AiRuiKaTM) is a high-affinity, selective, humanized immunoglobulin G4/k PD-1 monoclonal antibody. It interacts with PD-1 on immunity cells (including activated T lymphocytes, B cells, and natural killer cells) and programmed cell death ligand 2 (PD-L2) on antigen-presenting cells (Sharpe and Pauken, 2017). It was developed by Jiangsu Hengrui Medicine Co. Ltd. in China (Markham and Keam, 2019) and has been approved as a third-line treatment for relapsed or refractory classical Hodgkin lymphoma patients and second-line treatment for hepatocellular carcinoma (HCC) patients by the China Food and Drug Administration (Markham and Keam, 2019).

As a highly selective vascular endothelial growth factor receptor-2 (VEGFR-2) tyrosine kinase inhibitor, apatinib (YN968D1) has been approved as a third-line and subsequent treatment for advanced gastric adenocarcinoma or gastroesophageal junction adenocarcinoma in China (Zhang, 2015). The binding of VEGF and its receptors (VEGFR-1, VEGFR-2, or VEGFR-3) plays an important part in tumor-associated angiogenesis (Mi et al., 2010). Apatinib interacts with VEGFR-2, locks the VEGF signaling pathway, and thus inhibits tumor growth and metastasis (Scott et al., 2015). Besides, apatinib could promote immune response (Zhao et al., 2017; Zheng et al., 2018), overcome resistance to immunotherapy (Wang et al., 2020), and produce synergistic antitumor effects when combined with immunotherapy in the mouse model (Wang, et al., 2020). The combination has recently been reported in a few case reports (Yang et al., 2018; Zhao et al., 2019) and clinical trials (Liang et al., 2019; Liu et al., 2019; Xie et al., 2019).

So far, the safety and efficacy of camrelizumab and its combination with chemotherapy (and apatinib) have been assessed in patients with esophageal squamous cell carcinoma (ESCC), gastric cancer, HCC, nasopharyngeal carcinoma (NPC), non-small cell lung cancer (NSCLC), and so on. In addition, the optimal efficacy predictors, such as PD-L1 expression and tumor mutation burden (TMB), were mentioned in a few studies. In this study, we analyzed the safety and efficacy of camrelizumab and its combination in solid cancers, and determined the potential predictive biomarkers to accurately classify the most efficient patients based on published clinical trials.

## Method

### Literature Search

Studies were searched in Embase, PubMed, and the Cochrane Library databases with the keywords “camrelizumab” and “SHR1210” (publications up to February 31, 2020). The American Society of Clinical Oncology, European Society for Medical Oncology, World Conference on Lung Cancer, World Organization for Specialized Studies on Diseases of the Esophagus World Conference database, and Gastrointestinal Cancers Symposium were also searched for relevant publications. After excluding the duplicated articles, full-text articles and conference abstracts were reviewed by two reviewers (HY and KW) for eligibility independently. We solved all the disagreements by a discussion with the third author.

### Inclusion and Exclusion Criteria

Included articles had to satisfy the following criteria: 1) clinical trials concerning the safety or efficacy of camrelizumab or camrelizumab plus other drugs; 2) enrolled patients had pathologically confirmed solid cancers; 3) the safety or efficacy data were available; and 4) published in English.

The exclusion criteria were as follows: 1) studies were not related to camrelizumab or cancers; 2) studies lacked adequate safety or efficacy data; 3) studies enrolled less than ten patients; 4) studies conducted in hematological malignancy; and 5) retrospective studies, reviews, reports, comments, meta-analyses, letters, case reports, correction, or guideline.

### Data Extraction

The following data were extracted from the included articles: 1) the basic information of studies, including the first author, published year, clinical trial number, study design (including study phase), number of patients, cancer types, treatments, follow-up time, and so on; 2) adverse events (AEs) mentioned in at least two studies, including any grade and grade ≥3 treatment-related AEs; 3) efficacy data, such as overall response rate (ORR), disease control rate (DCR), duration of response, 6-month progression-free survival (PFS) rate, median PFS time, 12-month overall survival (OS) rate, and median OS time. To avoid duplication of data, we chose articles with more useful data rather than the most recent publications or those including more patients.

### Statistical Analyses

Statistical analyses were performed by Comprehensive Meta-Analysis V3 (Biostat, Englewood, NJ, United States) and Review Manager 5.3 (RevMan; The Cochrane Collaboration, Oxford, England) software. Safety was evaluated by calculating the proportion and derived 95% CI of any grade and grade ≥3 AEs in at least two studies. The efficacy of camrelizumab and the combination therapy was evaluated by calculating the proportion and derived 95% CI of the ORR, DCR, and pooled 6-month PFS rate. The odds ratio (OR) and corresponding 95% CIs were calculated to compare the ORR, DCR, and PFS rate. The hazard ratio (HR) and their 95% CIs were used to compare PFS and OS time. All statistical analyses were two-sided, and p values <0.05 were identified as statistically significant. A fixed-effects model was applied when inconsistency index (I^2^) <50% or else a random-effects model was used.

### Quality Evaluation

Systematic biases of the involved randomized controlled trials (RCTs) were evaluated using the Cochrane risk-of-bias tool (Review Manager 5.3). Non-randomized trials were evaluated according to the methodological index for non-randomized studies score (Slim et al., 2003). The scoring system has eight items for non-comparative studies, and each item is scored 0, 1, or 2. Score 0 means the studies did not mention an item; score 1 means the item was mentioned but not adequately; and score 2 means the item was adequately reported. Two researchers scored each trial for the risk of bias independently. All the disagreements were solved by a discussion with the third author.

## Results

### Study Selection

**FIGURE 1:**
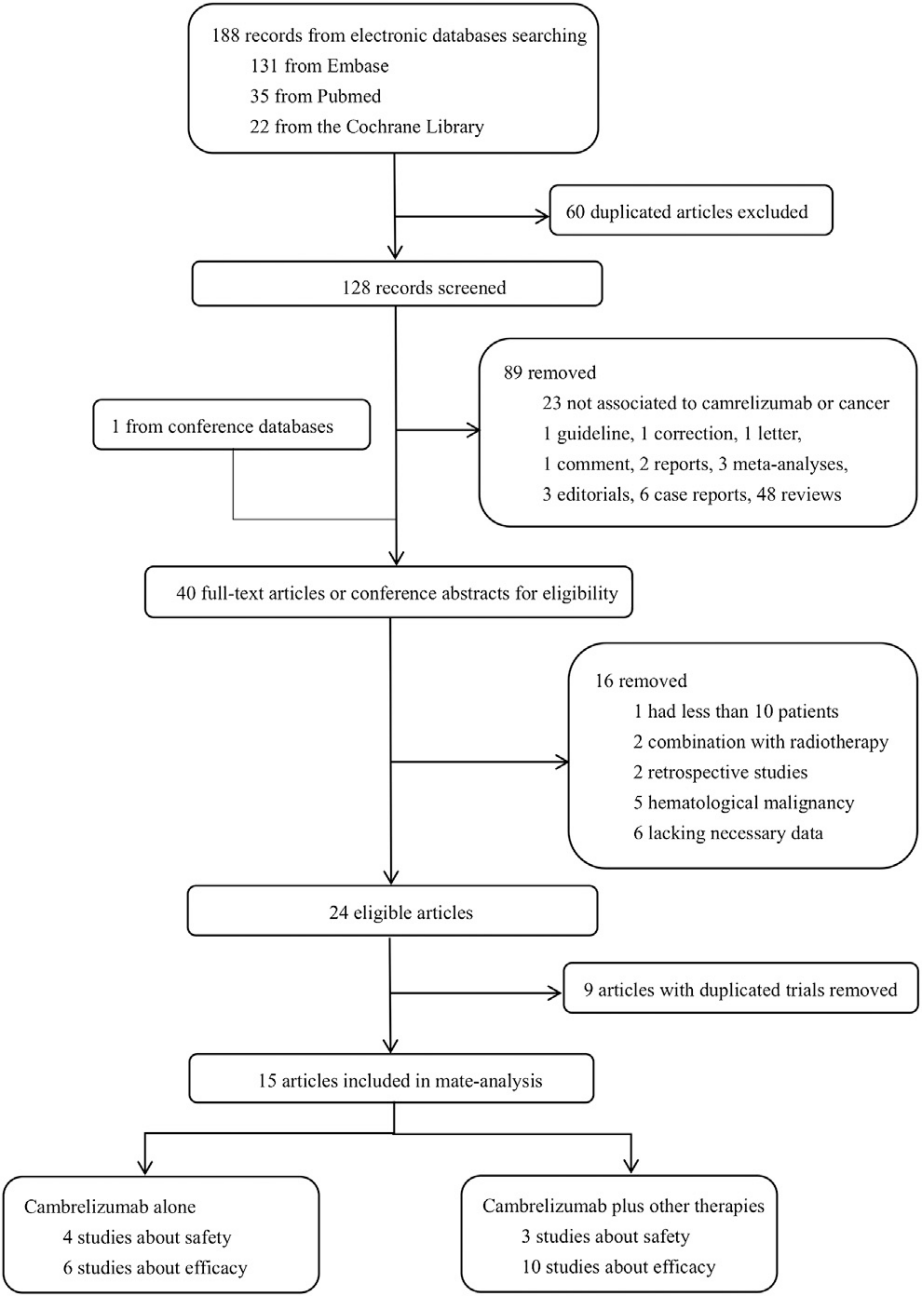
Progress of article selection.

A total of 189 articles were assessed. After carefully screening the full text or conference abstracts, 24 articles were eligible. Finally, 15 articles with 1,390 patients were included after removing nine articles with duplicate data (Fang et al., 2018; Huang et al., 2019a; Huang et al., 2019b; Liu et al., 2019; Qin et al., 2019; Shen et al., 2019; Wu et al., 2019; Xie et al., 2019; Xu et al., 2019; Zhang et al., 2019; Zhou et al., 2019a; Zhou et al., 2019b; Chen et al., 2020a; Lickliter et al., 2020; Qin et al., 2020). Trial NCT03121716 had both a single-agent cohort (camrelizumab alone) and a combination cohort (camrelizumab plus other therapies), and the two cohorts were enrolled separately as two studies (Fang et al., 2018). Trial NCT03394287 had a continuous cohort and an intermittent cohort (Liu et al., 2019). Patients received camrelizumab and apatinib for 14 days in the continuous cohort and were treated with only 7 days of apatinib in the intermittent cohort. Since only nine patients were evaluable in the intermittent cohort, we abandoned this cohort in our analysis. Finally, six studies on camrelizumab treatment and 10 studies on combination therapy were eligible, and the detailed selection procedure is shown in Figure 1.

### Study Characteristics

All eligible studies had ClinicalTrials.gov numbers. Among these studies, there were six phase I studies, seven phase II studies, one phase I/II studies, and two phase III studies. Most studies were published in 2019, three studies were published in 2020, and only two studies were published in 2018. Two studies were RCTs, and the other 14 articles were single-arm trials. Camrelizumab was administered intravenously at a dose of 200 mg every 2 or 3 weeks in most studies. Studies were conducted in patients with biliary tract cancer (BTC), ESCC, gastric cancer, HCC, NPC, NSCLC, osteosarcoma, and triple-negative breast cancer. The combination options were chemotherapy and apatinib. Basic information of the included studies is provided in Table 1.

**TABLE 1:** Basic information of included studies.

Study	Clinical trial number	Study design	Cancer type	Patients (n)	Treatment	Median follow-up time	Methodological index for non-randomized studies score
Cambrelizumab alone							
Fang et al. (2018)	NCT02721589	Phase I, multi-cohort, single arm	NPC	93	Camrelizumab (200 mg, q2w)	9.9 months	16
Huang et al. (2019b) –ESCC	NCT03099382	Phase III, RCT	ESCC	228	Camrelizumab (200 mg, q2w)	8.3 months	—
				220	Docetaxel or irinotecan	6.2 months	—
Huang et al. (2019a) – G	NCT02742935	Phase I, multi-cohort, single arm	Gastric or GEJ cancer	30	Camrelizumab (60–400 mg, q2w)	28.7 weeks	16
Wu et al. (2019)	NCT03085069	Phase I, single arm	NSCLC	146	Camrelizumab (200 mg, q2w)	NM	14
Lickliter et al. (2020)	NCT02492789	Phase I, single arm	Solid tumors	49	Camrelizumab (1–-10 mg/kg, q2w and 200 mg, q4w)	NM	14
Qin et al. (2020)	NCT02989922	Phase II, multi-cohort, single arm	HCC	217	Camrelizumab (3 mg/kg q2w and q3w)	12.5 months	16
Cambrelizumab plus other therapies							
Fang et al. (2018) – combination	NCT03121716	Phase I, multi-cohort, single arm	NPC	23	Camrelizumab (200 mg, q3w) + GC for 6 cycles followed by camrelizumab (200 mg, q3w)	10.2 months	16
Qin et al. (2019)	NCT3092895	Phase II, multi-cohort, single arm	HCC or BTC	81	Camrelizumab (3 mg/kg q2w) + FOLFOX4 or GEMOX	NM	14
Zhou et al. (2019a)	NCT03134872	Phase III, RCT	Non-squamous NSCLC with EGFR, ALK (−)	205	Camrelizumab (200 mg, q3w) + AC, followed by pemetrexed and camrelizumab	11.9 months	—
				207	AC, followed by pemetrexed	11.9 months	—
Chen et al. (2019a)	NCT03486678	Phase II, single arm	BTC	37	Camrelizumab (3 mg/kg, q2w) + GEMOX for 12 cycles followed by camrelizumab alone	NM	14
Liu et al. (2019)	NCT03394287	Phase II, multi-cohort, single arm	TNBC	24	Camrelizumab (200 mg, q2w) + apatinib (250 mg/day)	NM	14
Shen et al. (2019)	NCT03472365	Phase II, single arm	Gastric or GEJ cancer	48	Camrelizumab (200 mg, q3w) + CAPOX for 4–6 cycles followed by camrelizumab (200 mg, q3w) + apatinib (375 mg/day)	NM	14
Xie et al. (2019)	NCT03359018	Phase II, single arm	HOS	41	Camrelizumab (200 mg, q2w) + apatinib (500 mg/day)	NM	14
Xu et al. (2019)	NCT02942329	Phase I, multi-cohort, single arm	HCC, gastric, or EGJ cancer	43	Camrelizumab (200 mg, q2w) + apatinib (125-500 mg/day)	7.9 months	16
Zhou et al. (2019b)	NCT03083041	Phase I/II, multi-cohort, single arm	Non-squamous NSCLC with EGFR, ALK (−)	96	Camrelizumab (200 mg, q2w) + apatinib (250 mg/day)	22.1 months	16
Zhang et al. (2019)	NCT03603756	Phase II, single arm	ESCC	29	Camrelizumab (200 mg, q3w) + TP + apatinib (250 mg/day)	NM	14

AC, carboplatin + pemetrexed; BTC, biliary tract cancer; CAPOX, capecitabine + oxaliplatin; ESCC, esophageal squamous cell carcinoma; FOLFOX4, fluorouracil + leucovorin + oxaliplatin; GC, gemcitabine + cisplatin; GEMOX, gemcitabine + oxaliplatin; HCC, hepatocellular carcinoma; HOS, high-grade osteosarcoma; NPC, nasopharyngeal carcinoma; NSCLC, non-small cell lung cancer; TNBC, triple-negative breast cancer; TP, liposomal paclitaxel + nedaplatin.

### Overall Toxicity Analysis

**FIGURE 2:**
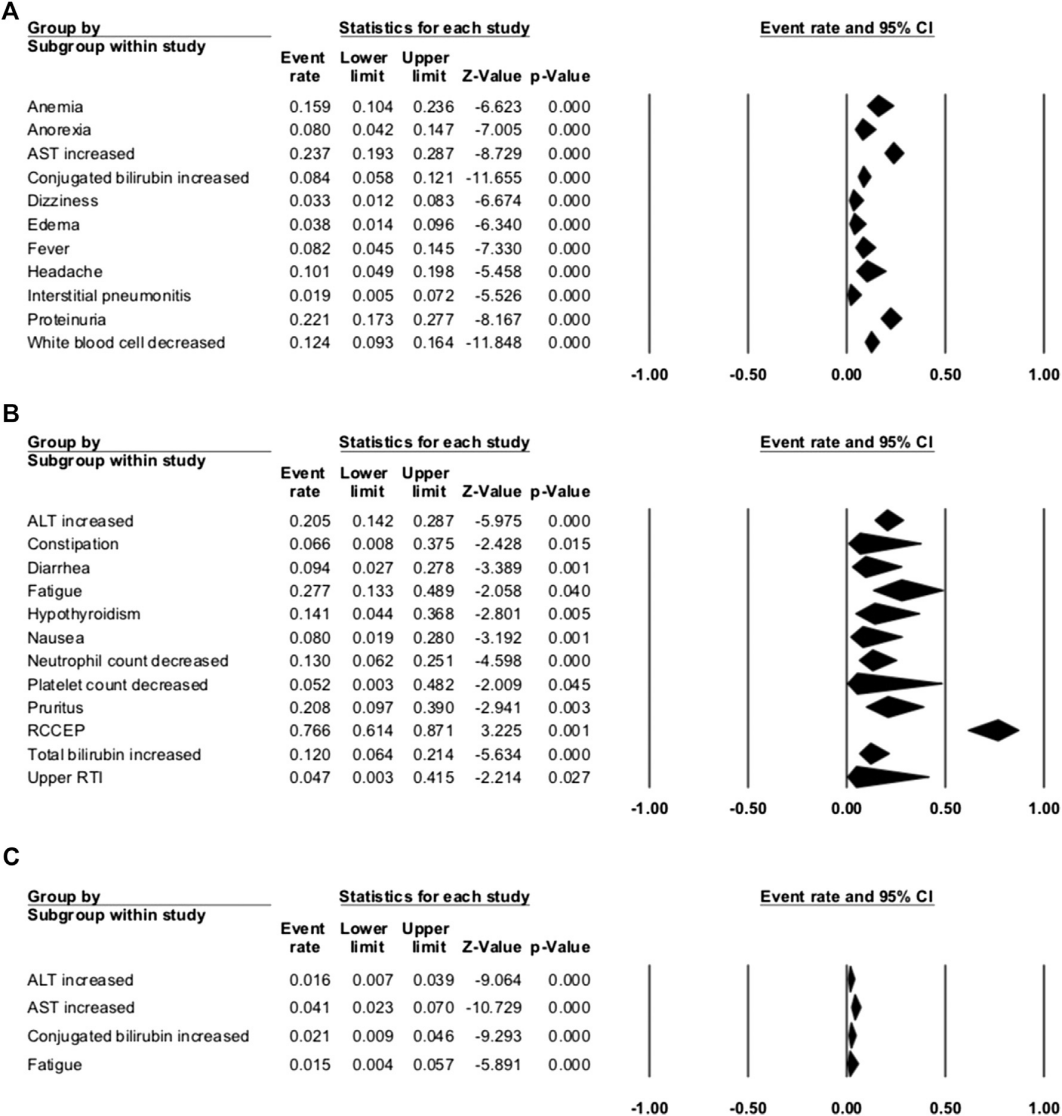
Adverse events (AEs) of camrelizumab alone. **(A)** The fixed model of all-grade AEs; **(B)** the random model of all-grade AEs; and **(C)** the fixed model of grade ≥3 AEs.

**FIGURE 3:**
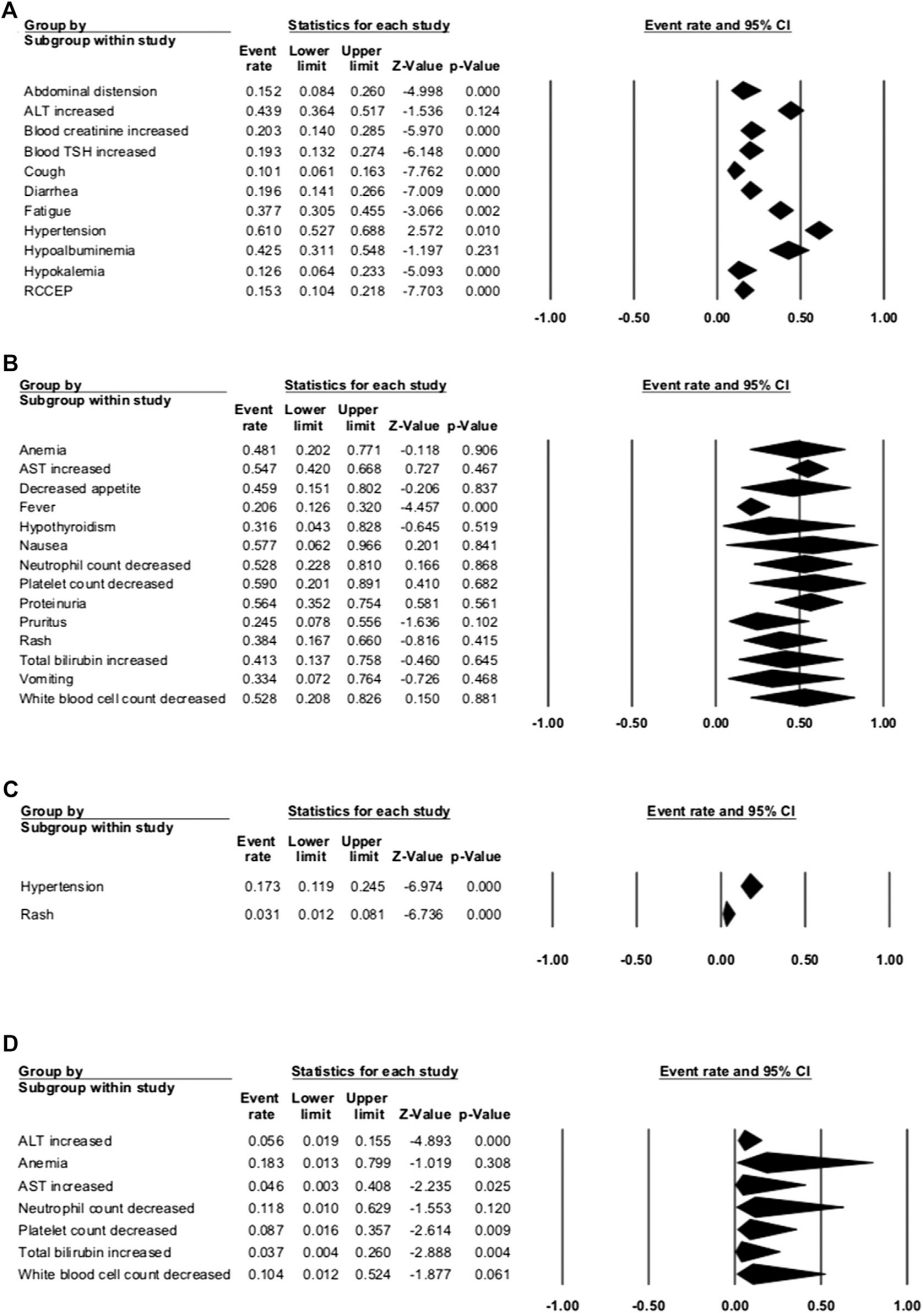
Adverse events (AEs) of combination therapy. **(A)** The fixed model of all-grade AEs; **(B**) the random model of all-grade AEs; **(C)** the fixed model of grade ≥3 AEs; and **(D)** the random model of grade ≥3 AEs.

Seven studies were enrolled to assess the AE rate, with three studies of combination therapy. The results are presented in Figures 2, 3. Figure 2 shows the result of camrelizumab treatment, including all-grade AEs (**A**, fixed model; **B**, random model) and grade ≥3 AEs (**C**, fixed model). Figure 3 shows the results of all-grade AEs in combination therapy (**A**, fixed model; **B**, random model) and the results of grade ≥3 AEs (**C**, fixed model; **D**, random model). Reactive cutaneous capillary endothelial proliferation (RCCEP) was the most common AE of camrelizumab treatment and occurred in 76.6% of patients. Hypertension ranked first in combination therapy and occurred in 61.0% of patients. Table 2 shows the top five most frequent AEs in the two groups, respectively.

**TABLE 2:** Top five most frequent any-grade AEs.

Cambrelizumab alone				Cambrelizumab plus other therapies			
AEs	Event rate (95% CI)	p value	Statistical method	AEs	Event rate (95% CI)	p value	Statistical method
RCCEP	0.766 (0.614–0.871)	0.001	Random	Hypertension	0.610 (0.527–0.688)	0.010	Fixed
Fatigue	0.277 (0.133–0.489)	0.040	Random	Platelet count decrease	0.590 (0.201–0.891)	0.682	Random
AST increase	0.237 (0.193–0.289)	0.000	Fixed	Nausea	0.577 (0.062–0.962)	0.841	Random
Proteinuria	0.221 (0.173–0.277)	0.000	Fixed	Proteinuria	0.564 (0.352–0.754)	0.431	Random
Pruritus	0.208 (0.097–0.305)	0.003	Random	AST increase	0.547 (0.420–0.668S)	0.881	Random

AEs, adverse events; RCCEP, reactive cutaneous capillary endothelial proliferation; AST, aspartate aminotransferase.

#### Camrelizumab Group

Among the enrolled articles, four studies were subsumed in any-grade AE analysis, and three articles were incorporated in the grade ≥3 AE analysis because some articles had no applicable data. The major AEs of any grade occurred in over 20% of patients with camrelizumab treatment, including RCCEP, fatigue, aspartate aminotransferase (AST) increase, proteinuria, pruritus, and alanine transaminase (ALT) increase. RCCEP was the most frequent AE with an incidence range from 61.2 to 88.2%, and the overall event rate was 76.6% (95% CI: 61.4–87.1%). Fatigue occurred in 27.7% (95% CI: 13.3–48.9%) of the patients, and AST increase occurred in 23.7% of the patients. Compared with the high incidence of any-grade AEs, the incidence of grade ≥3 AE was rare. The incidence of grade ≥3 AST increase was 4.1%. Other grade ≥3 AEs were conjugated bilirubin increase, ALT increase, and fatigue. These occurred in 2.1, 1.6, and 1.5% of the patients, respectively. Serious AEs that led to therapy discontinuation were reported in five studies, and treatment-associated death happened in two studies. The incidence of therapy discontinuation ranged from 3.7 to 7.7%, and death was reported in 0.3% of gastric cancer patients (one patient with interstitial lung disease) and 3.1% of ESCC patients (Table 3). Interstitial pneumonitis has a low incidence of 1.9% (95% CI: 0.5–7.2%), but it caused one death in gastric cancer patients. When using camrelizumab, doctors should take care of patients with poor lung function or interstitial lung disease.

**TABLE 3:** AEs leading to therapy discontinuation and deaths.

Study	Cancer type	Treatment	Discontinuation	Deaths
Fang et al. (2018)	NPC	Camrelizumab	7.7% Grade 3 subcutaneous hemorrhage (*n* = 1), grade 3 total bilirubin increase (*n* = 1), and grade 4 unconjugated bilirubin increase (*n* = 1).	0
Huang et al. (2019b) –ESCC	ESCC	Camrelizumab	7.0%	3.1%
Huang et al. (2019a) – G	Gastric or GEJ cancer	Camrelizumab	NM	0.3% Interstitial lung disease (*n* = 1)
Wu et al. (2019)	NSCLC	Camrelizumab	7.5%	NM
Lickliter et al. (2020)	Solid tumors	Camrelizumab	6.1%	0
Qin et al. (2020)	HCC	Camrelizumab	3.7% Abnormal hepatic function (*n* = 2), vascular rupture (n = 1), interstitial lung disease (*n* = 2), blood bilirubin increase and upper gastrointestinal hemorrhage (*n* = 1), increased γ-glutamyltransferase (*n* = 1), and increased ALT (*n* = 1)	0
Fang et al. (2018) –combination	NPC	Camrelizumab + GC	13.0% Pneumonitis (*n* = 1), rhinorrhagia (*n* = 1), and gastrointestinal reaction (*n* = 1) associated with chemotherapy	0
Qin et al. (2019)	HCC or BTC	Camrelizumab + FOLFOX4 or GEMOX	1.2% Recurrent grade 2 anemia related to FOLFOX4 (*n* = 1)	NM
Zhou et al. (2019a)	Non-squamous NSCLC with EGFR, ALK (−)	Camrelizumab + AC	NM	2.4%
Liu et al. (2019)	TNBC	Camrelizumab + apatinib	NM	0
Shen et al. (2019)	Gastric or GEJ cancer	Camrelizumab + CAPOX + apatinib	NM	0
Xie et al. (2019)	HOS	Camrelizumab + apatinib	NM	0
Xu et al. (2019)	HCC, gastric, or EGJ cancer	Camrelizumab + apatinib	4.7%	0

NPC, nasopharyngeal carcinoma; ESCC, esophageal squamous cell carcinoma; NSCLC, non-small cell lung cancer; HCC, hepatocellular carcinoma; ALT, alanine transaminase; GC, gemcitabine + cisplatin; BTC, biliary tract cancer; FOLFOX4, fluorouracil + leucovorin + oxaliplatin; GEMOX, gemcitabine + oxaliplatin; AC, carboplatin + pemetrexed; TNBC, triple-negative breast cancer; CAPOX, capecitabine + oxaliplatin; HOS, high-grade osteosarcoma.

#### Combination Group

In the three studies of combination therapy, combination with apatinib was used in two studies and combination with chemotherapy was used in one study. Among them, hypertension had the highest incidence of 61.0% (95% CI: 52.7–68.8%). Other common any-grade AEs occurred in more than 50% of the patients, including platelet count decrease (59.0%), nausea (57.7%), proteinuria (56.4%), AST increase (54.7%), and white blood cell count decrease (52.8%). The most common grade ≥3 AE was anemia, with the incidence of 18.3% (95% CI: 1.3–79.9%). Other grade ≥3 AEs that occurred in over 10% of patients were hypertension (17.3%), neutrophil count decrease (11.8%), and white blood cell count decrease (10.4%). AEs that led to therapy discontinuation were reported in three studies, and treatment-associated death happened in one study. The incidence of therapy discontinuation was 1.2–13.0%, and death happened in 2.4% NSCLC patients with camrelizumab and chemotherapy.

When comparing the incidence of any-grade AEs between camrelizumab alone and combination therapy, we found that most AEs occurred more frequently with combination therapy. The incidences of some AEs with camrelizumab alone were more than twice the incidences with combination therapy, like hepatic function (ALT, AST, or total bilirubin increase), hematologic toxicities (neutrophil, platelet, or white blood cell count), anemia, diarrhea, fever, nausea, and proteinuria. These were obviously associated with chemotherapy or apatinib. ALT increase and AST increase were common grade ≥3 AEs in both groups. This should remind doctors of the importance of hepatic function examination and liver-protective drugs during treatment. The incidence of RCCEP dramatically decreased with combination therapy (76.6 vs. 15.3%), ensured the safety of camrelizumab, and made this combination reasonable.

### Overall Efficacy Analysis

In our analysis, the pooled ORR, DCR, and 6-month PFS rate were used to judge the efficacy of camrelizumab and the combination therapy. Six studies of camrelizumab alone and 10 studies of combination therapy were tested.

#### Camrelizumab Group

**FIGURE 4:**
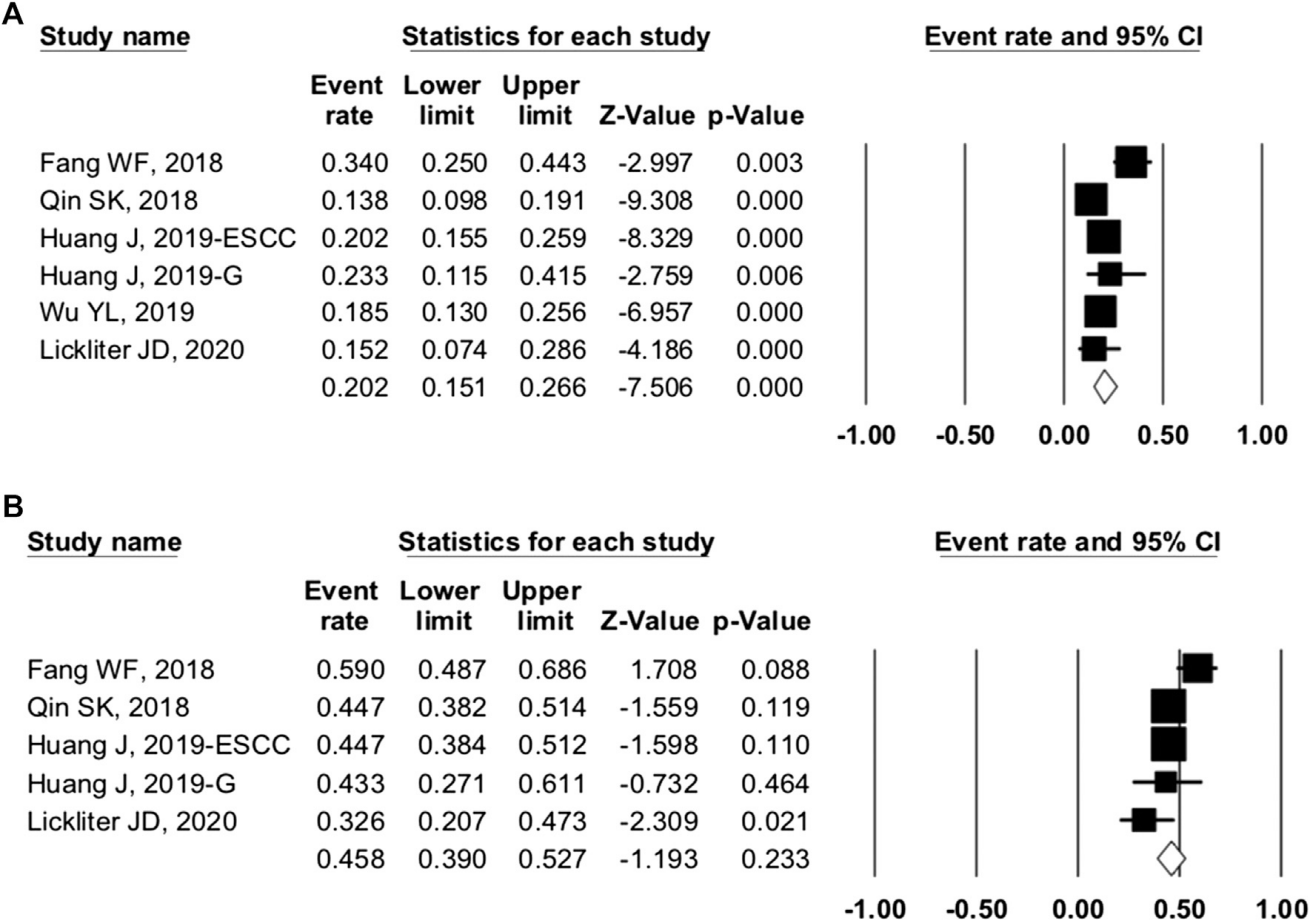
Efficacy of camrelizumab alone. **(A)** overall response rate and **(B)** disease control rate of included studies.

For camrelizumab alone, six studies were included in the ORR analysis, and five studies were enrolled in the DCR analysis. The pooled ORR and DCR were 20.2% (95% CI: 15.1–26.6%, p = 0.000, I^2^ = 70.360) and 45.8% (95% CI: 39.0–52.7%, p = 0.256, I^2^ = 58.661), respectively (Figure 4). The 6-month PFS rate was reported as 48.2% in trial NCT02721589 of NPC patients. The median TTR ranged from 1.83 to 1.9 months, the median PFS varied from 1.87 to 5.6 months, and the median OS varied from 8.3 to 19.4 months (Table 4).

**TABLE 4:** Efficacy data of enrolled studies.

Study	Cancer type	Evaluable patients	ORR	DCR	TTR (m)	DoR (m)	6-month PFS rate	PFS (m)	OS (m)
Cambrelizumab alone									
Fang et al. (2018)	NPC	91	0.340	0.590	1.9 (1.7–2.1)	NR (6.3–NR)	0.482	5.6 (3.3–7.9)	NR
Qin et al. (2020)	HCC	217	0.138	0.447	2.0 (1.7–6.2)	NR (2.0–15.4+)	—	2.1 (2.0–3.2)	NR
Huang et al. (2019b) – ESCC	ESCC	228	0.202	0.447	—	—	—	—	8.3 (6.8–9.7)
Huang et al. (2019a) –G	Gastric or GEJ cancer	30	0.233	0.433	1.83 (1.70–5.53)	8.43 (1.63–13.10+)	—	1.87 (1.83–1.90)	—
Wu et al. (2019)	NSCLC	146	0.185	—	—	15.1	—	3.2 (2.0–3.4)	19.4 (11.6–NR)
Lickliter et al. (2020)	Solid tumors	46	0.152	0.326	—	NR (1.2–NR)	—	1.9 (1.7–3.1)	—
Cambrelizumab plus other therapies									
Fang et al. (2018) –combination	NPC	23	0.910	1.000	1.6 (1.4–2.2)	NR	0.864	NR	NR
Liu et al. (2019)	TNBC	19	0.474	0.684	—	—	—	NR	—
Qin et al. (2019) – HCC	HCC	34	0.265	0.794	2.0 (1.5–5.7)	NR (3.3–11.5+)	—	5.5	NR
Qin et al. (2019) – BTC	BTC	43	0.070	0.674	1.9 (1.8–2.1)	5.3 (3.7–7.0)	—	NR	NR
Shen et al. (2019)	Gastric or GEJ cancer	43	0.442	0.767	—	NR	—	NR	—
Xie et al. (2019)	HOS	41	0.220	—	—	—	0.700	6.50 (4.23–7.50)	NR
Xu et al. (2019) – G	Gastric or EGJ cancer	25	0.174	0.783	2.8(1.4–6.0)	4.7	0.253	2.9 (2.5–4.2)	11.4 (8.6–NR)
Xu et al. (2019) – HCC	HCC	18	0.500	0.938	3.4 (1.4–9.7)	NR	0.454	5.8 (2.6–NR)	NR (4.0–NR)
Zhang et al. (2019)	ESCC	26	0.731	0.962	—	—	—	NR	NR
Zhou et al. (2019a)	NSCLC	205	0.600	0.873	—	17.6 (11.6–NR)	—	11.3 (9.5‒NR)	NR (17.1–NR)
Zhou et al. (2019b)	NSCLC	91	0.308	0.824	1.8 (1.8–5.5)	NR	—	5.9 (5.5–10.3)	NR
Chen et al. (2020a)	BTC	36	0.528	0.917	—	—	0.500	6.2 (4.2–7.1)	—

DCR, disease control rate; DoR, duration of response; m, month; NR, not reached; ORR, overall response rate; OS, overall survival; PFS, progression-free survival; TTR, time to response; NPC, nasopharyngeal carcinoma; HCC, hepatocellular carcinoma; ESCC, esophageal squamous cell carcinoma; NSCLC, non-small cell lung cancer; BTC, biliary tract cancer; HOS, high-grade osteosarcoma.

#### Combination Group

**FIGURE 5:**
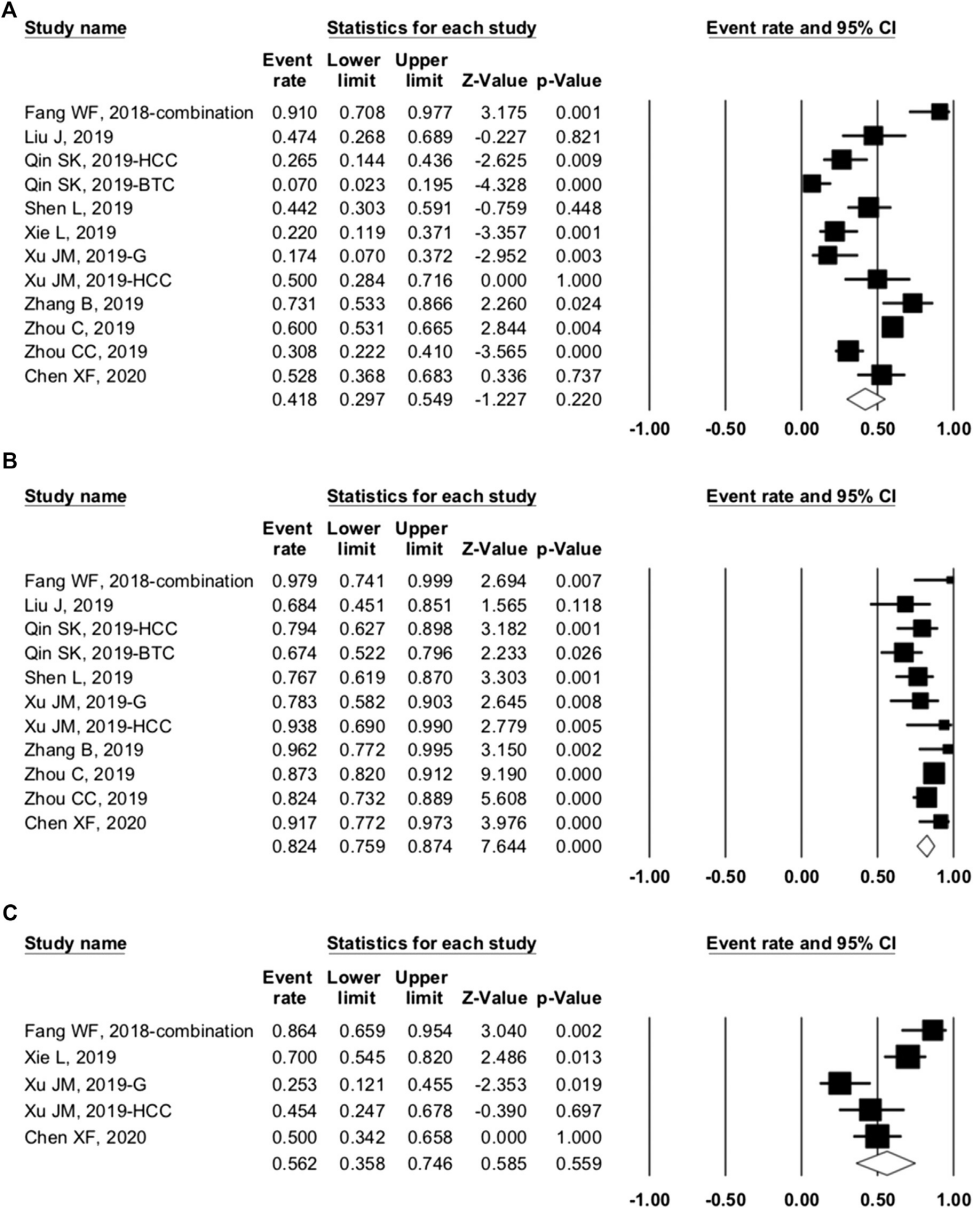
Efficacy of combination therapy. **(A)** overall response rate; **(B)** disease control rate; and **(C)** 6-month progression-free survival rate of included studies.

Ten studies were included in the efficacy analysis of combination therapy. The pooled ORR, DCR, and 6-month PFS rate were 41.8% (95% CI: 29.7–54.9%, p = 0.220, I^2^ = 86.265), 82.4 (95% CI: 75.9–87.4%, p = 0.000, I^2^ = 55.207), and 56.2% (95% CI: 35.8–74.6%, p = 0.559, I^2^ = 79.739) (Figure 5), respectively. So, the ORR and DCR were twice higher in patients with combination therapy than camrelizumab alone. Combination therapy appears to have better efficacy than camrelizumab alone, but a direct comparison between the two groups was lacking.

The efficacy data of two RCTs are displayed in Table 5. In the second-line treatment of ESCC, camrelizumab was better than chemotherapy in Chinese patients (NCT03099382). The camrelizumab group had better ORR (20.2 vs. 6.4%), better 12-month survival rate (33.7 vs. 22.3%), and longer OS (8.3 vs. 6.2 months, HR = 0.71, 95% CI: 0.57–0.87, p = 0.0010). NCT03134872 compared chemotherapy and camrelizumab plus chemotherapy as a first-line treatment in advanced or metastatic non-squamous NSCLC patients with negative epithelial growth factor receptor (EGFR) and anaplastic lymphoma kinase. The combination group showed superior ORR (60.0 vs. 39.1%) and PFS (11.3 vs. 8.3 months, HR = 0.61, 95% CI: 0.46–0.80, p = 0.0002).

**TABLE 5:** Efficacy data of two enrolled randomized controlled trials.

Efficacy data	Study	Treatment group	Control group	
Overall response rate	Huang et al. (2019b) – ESCC	0.202	0.064	—
	Zhou et al. (2019a)	0.600 (0.530–0.680)	0.391 (0.324–0.461)	p < 0.001
Disease control rate	Zhou et al. (2019)	0.873 (0.820–0.916)	0.744 (0.679–0.802)	p = 0.0009
Duration of response	Huang et al. (2019) – ESCC	7.4 (3.8–10.8)	3.4 (0.9–NR)	HR: 0.34 (95% CI: 0.14–0.92)
	Zhou et al. (2019)	17.6 (11.6–NR)	9.9 (8.5–13.6)	p = 0.0356
PFS (m)	Huang et al. (2019) – ESCC	1.9 (1.90–2.4)	1.9 (1.9–2.1)	HR: 0.69 (95% CI: 0.56–0.86, p = 0.0006)
	Zhou et al. (2019)	11.3 (9.5‒NR)	8.3 (6.0–9.7)	HR: 0.61 (95% CI: 0.46–0.80, p = 0.0002)
6-month PFS rate	Huang et al. (2019) – ESCC	0.216	0.045	—
OS (m)	Huang et al. (2019) – ESCC	8.3 (6.8–9.7)	6.2 (5.7–6.9)	HR: 0.71 (95% CI: 0.57–0.87, p = 0.0010)
	Zhou et al. (2019)	NR (17.1–NR)	20.9 (14.2–NR)	p = 0.0272
12-month OS rate	Huang et al. (2019) – ESCC	0.337	0.223	—

Note: Zhou et al. (2019) compared camrelizumab + chemotherapy vs. chemotherapy; Huang et al. (2019) – ESCC compared camrelizumab vs. chemotherapy. PFS, progression-free survival; OS, overall survival; HR, hazard ratio; ESCC, esophageal squamous cell carcinoma.

#### Subgroup Analysis

**FIGURE 6:**
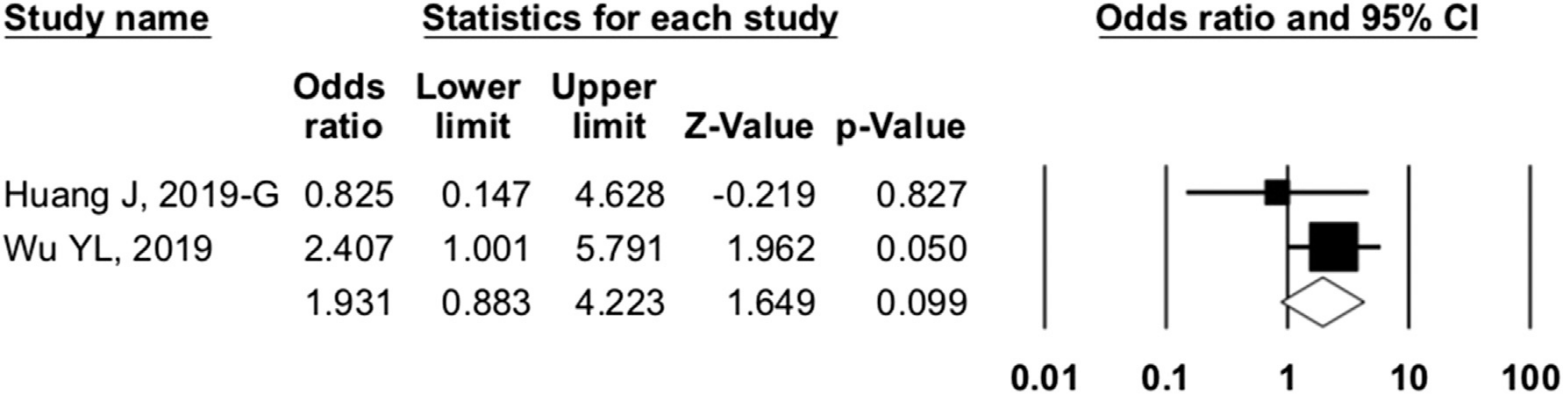
Comparison of programmed cell death ligand 1–positive and programmed cell death ligand 1–negative patients. Our study showed that PD-L1 expression (cutoff value: 1%) was not associated with overall response rate (odds ratio = 1.931, 95% CI: 0.883–4.223, p = 0.099).

To investigate the source of heterogeneity among studies, we conducted subgroup analyses. In trials of camrelizumab alone, NCT02742935 and NCT03085069 showed the ORR in different PD-L1 expression subgroups. Taking 1% as the cutoff value of PD-L1 positivity, we found the OR was 1.931 (95% CI: 0.883–4.223, p = 0.099) between PD-L1–positive and negative patients (Figure 6). Camrelizumab seems to have an effect regardless of PD-L1 expression, but this result had no significant difference.

In trial NCT02742935, elevated baseline lactate dehydrogenase (LDH) was associated with lower ORR (8.3 vs. 32.3%, p = 0.02), shorter PFS (1.8 vs. 4.0 months; HR = 0.39, p = 0.002), and shorter OS (4.2 vs. 10.2 months; HR = 0.22, p<0.0001) in ESCC patients (Wang et al., 2019). In another trial of advanced BTC patients (NCT03486678), increased baseline LDH level was also associated with poor PFS (5.0 vs. 6.2 months, p = 0.053) and shorter OS (6.8 vs. 12.6 months, p < 0.001) (Chen et al., 2020b). Besides, BTC patients with high TMB (cutoff value: 8.6 muts/Mb) had a significantly higher ORR (100 vs. 26%, p = 0.0294) (Chen et al., 2019b; Wu et al., 2019). In NSCLC patients treated with camrelizumab and apatinib, high TMB (cutoff value: 1.54 muts/Mb) was associated with higher ORR (52.6 vs. 17.1%) and better PFS (7.8 vs. 5.2 months). Of note, for HCC patients treated with camrelizumab, patients who developed RCCEP had better objective response (19·3 vs. 5.6%) (Qin et al., 2020).

**FIGURE 7:**
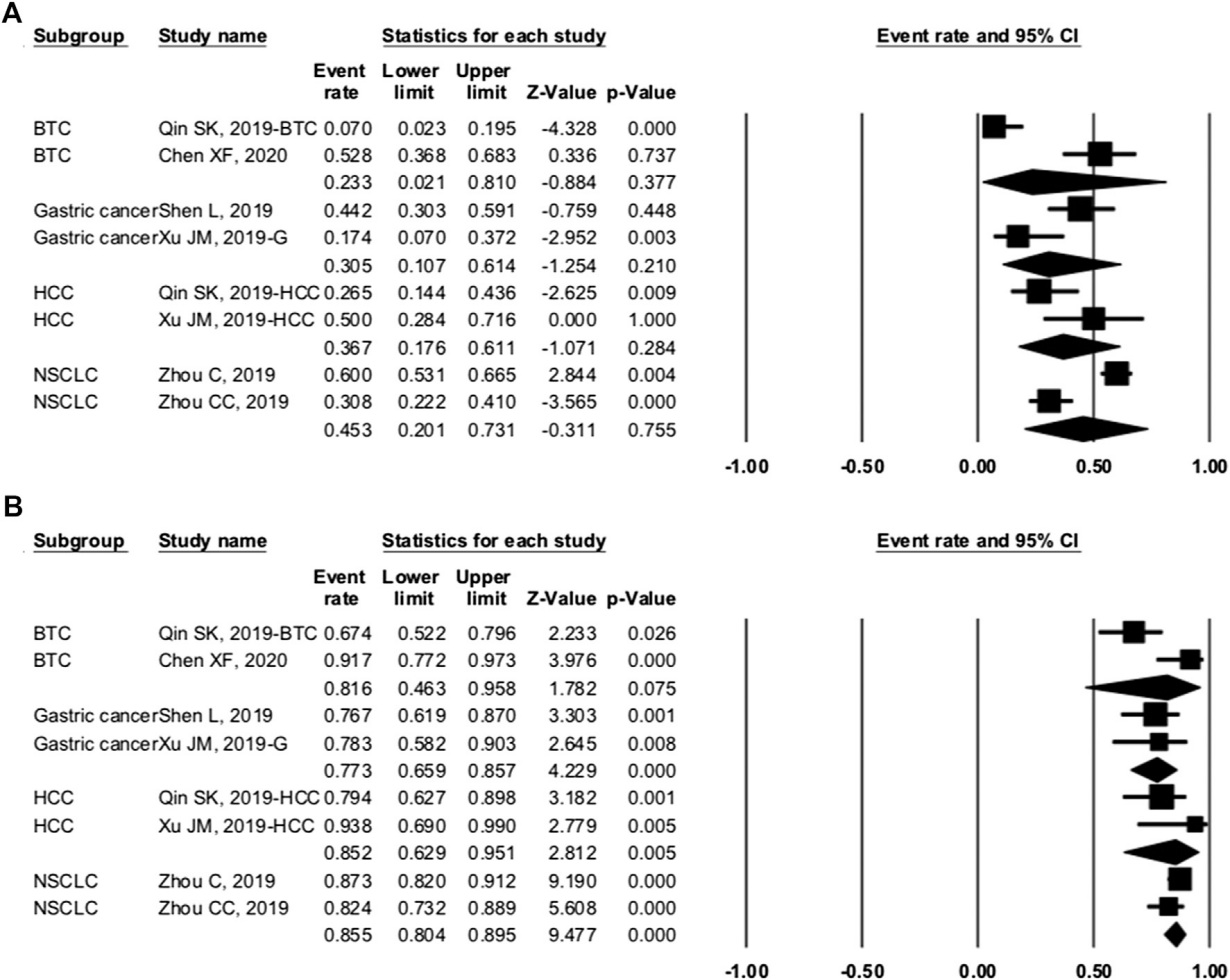
Subgroup analysis of cancer types. **(A)** overall response rate and **(B)** disease control rate of different cancers.

We grouped trials with combination therapy by cancer types (Figure 7). The pooled ORR of BTC, gastric cancer, HCC, and NSCLC was 23.3% (95% CI: 2.1–81.0%, p = 0.377), 30.5% (95% CI: 10.7–61.4%, p = 0.210), 36.7% (95% CI: 17.6–61.1%, p = 0.284), and 45.3% (95% CI: 20.1–73.1%, p = 0.755), respectively. The DCR was 81.6% (95% CI: 46.3–95.8%, p = 0.075), 77.3% (95% CI: 65.9–85.7%, p = 0.000), 85.2% (95% CI: 62.9–95.1%, p = 0.005), and 85.5% (95% CI: 80.4–89.5%, p = 0.0.000), respectively. When grouped by treatment frequency, the pooled ORR of treatment every 2 weeks and every 3 weeks was 30.2% (95% CI: 20.4–42.2%, p = 0.002) and 65.4% (95% CI: 48.5–79.1%, p = 0.074), respectively. The DCR of the two groups was 79.4% (95% CI: 71.3–85.7%, p = 0.000) and 87.5% (95% CI: 76.2–93.8%, p = 0.000), respectively. Data were insufficient for the analysis of PFS, OS, and others.

### Assessment of Study Quality and Publication Bias

**FIGURE 8:**
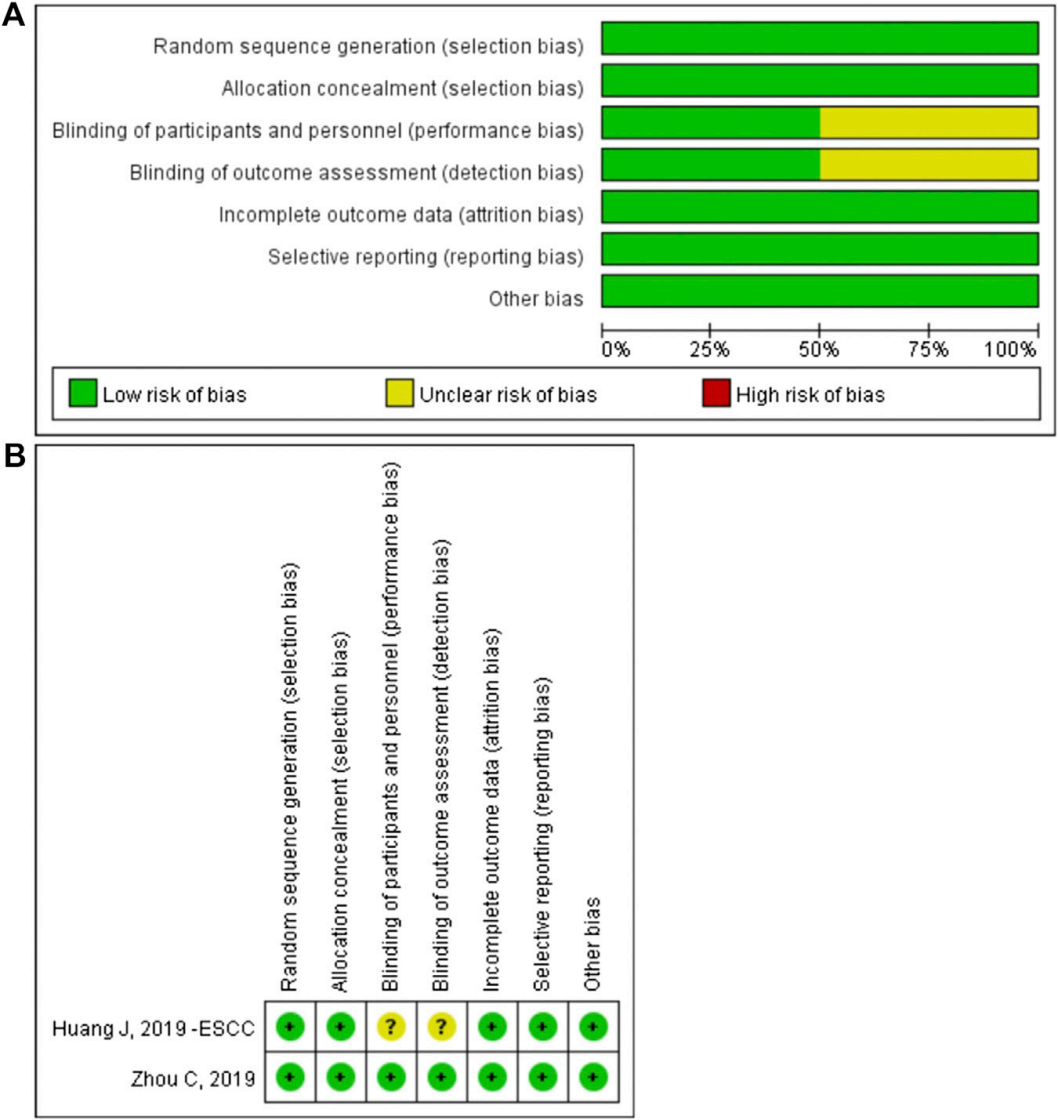
The risk of bias graph **(A)** and the risk of bias summary **(B)**.The overall risk of bias was evaluated as low risk.

The methodological quality of the enrolled RCT study was assessed by Review Manager 5.3. The risk of bias graph and risk of bias summary are shown in Figure 8. The non-randomized studies were assessed by the methodological index for non-randomized studies score (Table 1).

## Discussion

Camrelizumab was the first ICI independently developed by Chinese biopharma. It has been approved for classical Hodgkin lymphoma and HCC patients in China (Markham and Keam, 2019). The recommended dose is 200 mg *via* intravenous infusion once every 2 weeks until intolerable toxicity or disease progression occurs. This study investigated the efficacy and safety of camrelizumab and its combination in solid cancers.

As we know, ICIs could enhance the nonspecific immune response of hosts. In our pooled analysis, the most common AEs for camrelizumab alone were RCCEP, fatigue, AST increase, proteinuria, and pruritus. RCCEP has been considered as a unique toxicity of camrelizumab treatment (Chen et al., 2019a; Teng et al., 2019), but it has also been reported in 2.4% of patients treated with nivolumab and pembrolizumab for advanced melanoma (Hwang et al., 2016). In our study, RCCEPs occurred in as high as 76.6% of various solid cancer patients, and there was no grade ≥3 RCCEPs. The median time from the camrelizumab treatment to the onset of RCCEP was 2–4 weeks in previous studies (Chen et al., 2019a; Chen et al., 2020a). Most RCCEPs occurred on the skin (mainly on the face and trunk), and a few were found in oral and nasal mucosa. RCCEPs were mild and self-limiting. Apparent involution and complete regression of RCCEP could be observed both during and after treatment. Treatment was not necessary in most cases, except for patients with a high risk of bleeding.

The probable mechanism of RCCEP remains unclear now. The binding epitope of camrelizumab differs from other PD-1 inhibitors, which might influence the regulation of PD-1 signaling (Qin et al., 2020). A study reported that camrelizumab can increase VEGF expression on human umbilical vein endothelial cells and promote the proliferation and migration of human umbilical vein endothelial cells by activating hypoxia-inducible factor-1 α (HIF-1 α)/VEGF pathway *in vivo* (Wu et al., 2020). Another study performed human receptor proteome screening and identified that camrelizumab has a low affinity, highly selective interaction with VEGFR-2 (Finlay et al., 2019). The off-target binding and agonism of VEGFR-2 drive angiogenesis *via* vascular endothelial cell activation and lead to an abnormal proliferation of cutaneous capillary endothelial cells. When combined with apatinib, the anti-angiogenesis function of apatinib dramatically helps decrease the incidence of RCCEP to 15.3% and further ensures the safety of camrelizumab in combination therapy.

Meanwhile, apatinib has some common toxicity, including hypertension, hand–foot syndrome, proteinuria, and hematologic toxicity in treating solid cancers. Hypertension is the most common AE in combination therapy and is thought to be associated with the anti-angiogenesis function of apatinib on the normal vasculature. In previous studies of apatinib alone, incidences of hypertension and grade ≥3 hypertension were about 40.0 and 10.0%, respectively, in advanced gastric cancer patients (Li et al., 2013; Yang et al., 2019). It mostly occurred in 2 weeks of treatment and was controllable. The incidence of hypertension increased to 61.0% after combining with camrelizumab. Blood pressure should be actively monitored during and after patients received combination treatment. Once it happens, antihypertensive drugs should be used to avoid treatment disruption.

Proteinuria always occurred 3 weeks after apatinib treatment, with an incidence of about 50.0% in gastric patients (Li et al., 2016). The incidence was 56.4% in patients with combination treatment in our study, which was similar to the previous report. Routine urinalysis should be performed every 2 weeks to monitor proteinuria. If it occurs, dose decrease or treatment suspension may be required.

Both apatinib and camrelizumab can cause hematologic toxicity and hepatic function abnormality. Hepatic function abnormality could manifest as ALT elevation, AST elevation, or an increase in total bilirubin. The incidences of associated AEs with combination therapy were more than twice the incidences of associated AEs with camrelizumab alone. Routine blood and liver function examination tests at least every 2 weeks are necessary to timely detect the AEs.

For camrelizumab alone, the pooled ORR and DCR were 20.2 and 45.8%, respectively. The median PFS varied from 1.87 to 5.6 months, and the median OS was 8.3 to 19.4 months. According to NCT03099382, camrelizumab was better than chemotherapy in Chinese patients with ESCC (Huang et al., 2019b; Xu et al., 2019). The camrelizumab group showed better ORR and longer PFS and OS. More RCTs in different cancers would further ensure the efficacy of camrelizumab. The ORR and DCR of combination therapy increased to 41.8 and 82.4%, respectively, which were twice of those of camrelizumab alone. The combination of camrelizumab and apatinib is safe and appears to have better efficacy than camrelizumab alone. Future trials will provide further evidence.

To investigate the source of heterogeneity among studies, we conducted subgroup analyses. In studies of camrelizumab alone, PD-L1 expression was not associated with better ORR. Taking 1% as the cutoff value of PD-L1 positivity, we found the OR was 1.931 (95% CI: 0.883–4.223, p = 0.099) between PD-L1–positive and negative patients (Figure 6). However, there were only two associated trials, and further studies are required. Other possible predictive biomarkers included TMB, LDH, and the incidence of RCCEP. The association between these biomarkers and prognosis needs to be further studied.

In the enrolled cancer patients receiving combination therapy, the ORR ranged from 45.3% for NSCLC to 23.3% for BTC (Figure 7). The DCR varied between 77.3 and 85.5%. NSCLC patients had the highest ORR and DCR in our study. When grouped by treatment frequency, the pooled ORR was higher in treatment every 3 weeks (65.4 vs. 30.2%) (Figure 8). The DCRs were prominent in both groups (79.4 vs. 87.5%). Regardless of whether the patients received camrelizumab every 2 or 3 weeks, both frequencies could provide clinical benefits.

In this meta-analysis, several limitations are unavoidable. First, patients included in this study were heterogeneous with different cancer types and stages. The incidence of AEs may be totally different in different cancers. Second, the enrolled clinical trials used different doses, frequencies, and treatment lines of camrelizumab. These factors may affect the incidence of AEs and efficacy data. Third, some trials were published as conference abstracts and introduced some biases to the analysis. Most trials reported ORR, DCR, or PFS instead of OS because of insufficient follow-up time. A large meta-analysis revealed that PFS was not a surrogate endpoint for OS (Cortazar et al., 2014; Prasad et al., 2015; Paoletti et al., 2020). Evidences for the efficacy of camrelizumab were unsatisfactory without OS data. Finally, most of the included studies were single-arm studies without enough double-blinded RCTs, and some trials were in phase I or II. The failure of olaratumab in the treatment of soft tissue sarcoma showed the inconformity of phase II and phase III clinical trials (Pontes et al., 2020). A lot of new medicines were unable to translate promising phase II results into positive results in phase III trials. The effectiveness of camrelizumab also must be tested and verified in phase III comparative confirmatory trials. Considering those uncertainties in trials, a larger homogeneous patient pooled analysis and more phase III confirmatory trials with more survival results are needed to verify our conclusions.

## Conclusion

Camrelizumab and its combination with chemotherapy and apatinib appeared to have durable efficacy with tolerable toxicity in patients with various solid cancers. Combination therapy appeared to provide more clinical benefit with increasing AEs. However, these remain to be proven in future studies. RCCEP is the most common AE in camrelizumab treatment, and the incidence appears to appreciably decrease after combining with apatinib. Nevertheless, the incidences of hypertension, proteinuria, and hepatic function abnormality should be noted and regularly monitored during and after treatment. Baseline LDH may be a predictor of efficacy, and the role of PD-L1 and TMB, and the incidence of RCCEP may be clarified in further studies.

## Author Contributions

HY generated the idea. KW and BL collected the data and wrote the manuscript. HY and LY analyzed the data. ML and LY helped in correcting the grammar and editing the pictures. All authors read and approved the final version of the manuscript.

## Funding

This work was supported by the Science and Technology Department, Henan Province (grant numbers: 152300410164, 192102310048, and SB201901113).

## Conflict of Interest

The authors declare that the research was conducted in the absence of any commercial or financial relationships that could be construed as a potential conflict of interest.
